# Editorial: Molecular Mechanisms and Signaling in Endothelial Cell Biology and Vascular Heterogeneity

**DOI:** 10.3389/fcell.2021.821100

**Published:** 2021-12-17

**Authors:** Bin Ren, Ramani Ramchandran, Xiaofeng Yang

**Affiliations:** ^1^ Department of Surgery, O’Neal Comprehensive Cancer Center, and Comprehensive Cardiovascular Center, Heersink School of Medicine, University of Alabama at Birmingham, Birmingham, AL, United States; ^2^ Department of Pediatrics and Children’s Research Institute, Medical College of Wisconsin, Milwaukee, WI, United States; ^3^ Department of Cardiovascular Sciences, Centers for Cardiovascular Research and Metabolic Disease Research, Lewis Katz School of Medicine at Temple University, Philadelphia, PA, United States

**Keywords:** endothelial cells, angiogenesis, arterial, lymphangiogenesis, signaling, differentiation

Early in 1865, the Swiss anatomist Wihelm His introduced a new concept of *endothelium* in a programmatic assay and first defined the endothelium as the lining of the vasculature and the lymphatic system (Aird, 2007). Endothelium was considered as a cover and barrier facing a hollow face. The blood and lymphatic vessels are always filled with blood, different types of cells and other substances. Endothelial cells (ECs) may actually mediate communications between biological substances in the circulation system and other tissues. Later in 1958, Hibbs et al. (1958) noted that there was some variation in the structure of capillaries and arterioles from one organ to another, and even among blood vessels of the same organ . It is reasonable to speculate that the heterogeneity of vascular ECs may contribute to this difference in the structure and functional variations.

Nevertheless, with the first successful isolation and primary culture of human ECs from umbilical veins independently by Jaffe et al. (1973a) and Jaffe et al. (1973b) and Gimbrone et al. (1974), extensive studies on EC biology and growth of new blood vessels have started at molecular and cellular levels and by using animal models and human patient samples. Along with the discovery of tools for the culture of vascular and lymphatic ECs and other new models and technologies, the concept of the heterogeneity of ECs and blood and lymphatic vessels is getting increased recognition as an important feature of vascular biology by several researchers and scientists in various disciplines of biomedical research (Aird, 2006; Kang et al., 2010; Yuan et al., 2016; Mojiri et al., 2019; Ren et al., 2019; Takeda et al., 2019; Turgeon et al., 2020; Xiang et al., 2020; Sibler et al., 2021). It is known that ECs can interact with the blood constituents such as RBCs and can also interact with cells in a tissue that they reside in to influence functions (Cao et al., 2017; Kumar et al., 2019; Akil et al.; Jiang et al., 2021). Despite immense progress in our understanding of the molecular mechanisms and signaling that regulate EC patterning, the organ-specific function of ECs and the underlying mechanisms that dictate organ-specific function is poorly understood. In this special topic of “*Molecular Mechanisms and Signaling in Endothelial Cell Biology and Vascular Heterogeneity*,” researchers from a variety of institutions have submitted manuscripts that report EC function in various tissue beds, which influence vascular development process in health and disease. We received two hypothesis articles, ten original research articles and thirteen review articles for a total of 25 original contributions to this research topic. These articles cover many areas including EC heterogeneity, mechanisms underlying EC heterogeneity, novel ligands and receptor signaling pathways in vascular biology, angiogenesis, and lymphangiogenesis in health and disease. We will discuss these aspects and hope to provide insights into EC and vascular biology in this editorial.

## Ligands, Receptor and Intracellular Signaling in ECs

We begin with vascular endothelial growth factor (VEGF), also known as vascular permeability factor (VPF), one of the most important growth factor in EC and vascular biology. This important proangiogenic molecule was discovered over 3 decades ago by the Dvorak Laboratory at Harvard Medical School (Senger et al., 1983) and was first isolated and cloned by Napoleone Ferrara and his colleagues at Genentech 1989 (Leung et al., 1989). In an hypothesis article (Dvorak), Dr. Dvorak describes that VEGF is widely believed to induce angiogenesis by its direct mitogenic and motogenic actions on vascular ECs. However, he emphasizes the role of VEGF in vascular permeability, the role of delayed hypersensitivity in pathological stroma generation, particularly extravascular fibrin deposition and clotting within the tumor microenvironment (TME). Based upon his prior work where he concludes that tumors are “wounds that do not heal,” he now proposes that tumors are wounds that continually exhibit elements of local healing but do damage the host (patients) whereas the healing process actually facilitates tumor survival and growth. Moreover, solid tumors, and healing wounds feature enlarged feeder arteries, draining veins, and several other abnormal vessel types in addition to new angiogenic blood vessels. He also proposes that that VPF/VEGF induces stroma formation primarily by way of its potent “VPF” function. Much remains to be explored about the signaling and functions of VEGF in wound healing, tumor progression and chronic inflammatory diseases.

VEGF generally signals through interaction with its receptors. An article reviewed key advances in our understanding of VEGF signaling via VEGF receptor 2 (VEGFR-2) (Simons et al., 2016), a predominant receptor that mediates angiogenesis and arteriolar differentiation. In this special topic, Wang et al. describe the basics of VEGF/VEGFR-2 signaling and provide an update on VEGFR-2 signaling-mediated physiological and pathological functions as well as potential treatment strategies. Potent VEGF signaling also promotes angiogenesis and dictates arterial fate but inhibits venous specification, and this may depend on Notch signaling status (Lanner et al., 2007; CasieChetty et al., 2017). Akil et al. provide a comprehensive overview and update about Notch signaling in EC functions and angiogenesis. They emphasize the critical role and mechanisms by which Notch signaling promotes tumor progression via stimulating tumor angiogenesis, inducing phenotypes of cancer stem cells (CSCs) and thus conferring therapeutic resistance. They also discuss the Notch signaling crosstalk between CSCs and vascular ECs within the TME. They believe that therapeutic approaches could be developed by targeting the essential role of Notch pathway in CSCs and development of the arteriolar niche that promotes the self-renewal of CSCs (Jiang et al., 2021).

External stimuli induce a specific intracellular signaling pathway that can often come from multiple ligands. To integrate these multiple signaling pathways, adaptor proteins can ensure the temporal and spatial regulation of cellular signaling and contribute significantly to the signaling specificity. Cui et al. (2021) provide an excellent and focused review on endocytic adaptors epsins and Dab2 in the context of cardiovascular disease. Additionally, Alfaidi et al. emphasize the role of Nck1/2 adaptor proteins in vascular biology, vascular permeability and angiogenesis, and discuss their therapeutic potential. A study on an adaptor protein widely expressed in vascular ECs by Liu et al. demonstrates that the mitogen-inducible gene 6 acts as a potent anti-angiogenic factor in the regulation of physiological and pathological angiogenesis. In terms of anti-angiogenesis, thrombospondin-1 (TSP-1) is one of the first discovered endogenous angiogenesis inhibitor, and negatively regulates EC functions (Jiménez et al., 2000; Ren et al., 2006; Ren et al., 2009). Morandi et al. describe about new mechanisms by which the presence of TSP-1 at the plasma membrane affects EC signal transduction and angiogenesis via induction of receptor clustering. Additionally, small GTPase Rap1 is essential for the maintenance of EC homeostasis by promoting NO release. Rap1A and Rap1B are closely related Rap1 isoforms and regulate vascular homeostasis (Chrzanowska-Wodnicka, 2017). Kosuru et al. show that Rap1B directly and positively regulates eNOS activation whereas Rap1A prevents negative regulation of eNOS, thereby converging NO release, which may be important in the regulation of hypertension.

## EC Function in Development and Disease

EC function is intimately associated with developmental processes and dysfunction of ECs is associated with several disease states. For example, arterial specification of vascular ECs is essential for arteriogenesis. Chen et al. discuss the arterial fate specification from a developmental perspective (Chen et al.). Although VEGF-activation of Notch signaling is known as the key to arterial specification, they have performed carefully analysis in the settings of *de novo* vasculogenesis of the dorsal aorta during early embryogenesis and vasculature development in the neonatal mouse retina and described novel signaling mechanisms and a new understanding of this subject. They also emphasize the role of shear stress in the maintenance of arterial identity after blood circulation is established, as shear-induced Notch signaling activation and cell cycle arrest may contribute to A/V specification process (Fang et al., 2017). However, much remains to be investigated as for how arteriolar networks are involved under pathological ischemic conditions. In this regard, Nguyen et al. demonstrate that the deficiency of endothelial aryl hydrocarbon receptor nuclear translocator may exacerbate impaired angiogenic potential under peripheral ischemic conditions in mice with type 2 diabetes, which may be associated with defective angiogenic activity of ECs due to oxidative stress. As persistent oxidative stress during diabetes contributes to coronary endothelial dysfunction, and reactive oxygen species (ROS) mainly originates from the mitochondria in diabetes, a study from Xing et al. (2021) demonstrates that chronic inhibition of mitochondrial ROS may improve coronary endothelial function/dilation and SK channel activity in an animal model of diabetes. Furthermore, Zhao et al. describe the paradox of adenosine monophosphate-activated protein kinase (AMPK), a heterotrimeric serine-threonine kinase, in the involvement of vascular remodeling and the development of pulmonary hypertension. They emphasize the differential effects of AMPK on pulmonary vasoconstriction and pulmonary vascular remodeling, which may also be involved in the regulation of pathological microvascular remodeling and angiogenesis under obese conditions (Dong et al., 2017).

## EC Heterogeneity

EC heterogeneity is emerging as an important area in vascular biology. It is well known that ECs show tissue- and organ-specificity, and significantly contribute to the formation of different types of blood and lymphatic vessels. They serve as an important cell type in the development of vasculogenesis, angiogenesis, venogenesis and arteriogenesis under physiological and pathological conditions. As newly classified innate immune cells (Drummer et al., 2021; Shao et al., 2020; Mai et al., 2013), ECs are essential for normal tissue homeostasis and various pathologies (Aird, 2006; Ren et al., 2019; Marcu et al., 2018; Miyasaka, 2021) including contribution to the pathogenesis of COVID-19 (Shao et al., 2021). Majority of articles in this topic focus on EC heterogeneity and biology in health and disease. Dawson et al. review the heterogeneity of EC types, states, and phenotypes based upon the findings from ECs in multiple different experiments and among several tissue types and disease states and by using new techniques in transcriptome analysis, particularly single cell RNA-sequencing (also see single Cell Portal at https://singlecell.broadinstitute.org/single_cell). They functionally classify ECs as quiescent ECs involved in maintaining homeostasis, proliferative ECs, inflammatory ECs, remodeling ECs, ECs involved in EndMT, and ECs involved in angiogenesis. This classification may well explain two new connected processes such as inflammatory angiogenesis and non-inflammatory regenerative angiogenesis dissected in hind-limb ischemia-triggered angiogenesis (Fu et al., 2020). Dawson et al. also provide an update on innate contributions to EC heterogeneity, effects of biomechanical and biochemical stress on ECs and EC phenotypes in cardiovascular disease. Interestingly, Zhao et al. characterize the phenotypic and metabolic heterogeneity of ECs in diabetes-associated atherogenesis at the single-cell level by performing single-cell RNA sequencing study using EC-enriched single cells from mouse heart and aorta. Their study provides critical insight into EC biology and EC-related cardiovascular diseases. Whereas, Kim et al. provide an overview on the angiocrine role of heterogeneous subsets of cardiac ECs during cardiac development, shedding lights on the heterogenic nature of angiocrine signaling within the cardiac arterial, venous, and lymphatic ECs. In addition, Han et al. update the bone morphogenetic protein signaling functions in the regulation of EC heterogeneity and the underlying molecular mechanisms. Furthermore, Fang et al. present a comprehensive overview about regulation of endothelial functions by acetylation of histone proteins, a fundamental process that regulates gene expression epigenetically. They also discuss the roles of histone acetylation in ECs under physiological and pathophysiological conditions including vascular tone, inflammation, oxidative stress, angiogenesis, barrier function, thrombosis, and coagulation. An interesting study (Xia et al.) shows that lipopolysaccharide induces transcriptional activation of forkhead box protein C2 and promotes itself expression in a histone acetylation manner using lung ECs and a sterile sepsis model in neonatal mice.

## Novel Mechanisms Underlying Sprouting Angiogenesis and Lymphangiogenesis

In the field of angiogenesis, the endothelial-to-mesenchymal transition (EndoMT/EndMT) and partial epithelial-to-mesenchymal transition (EMT)/EndoMT have been extensively reported. EndoMT is a process whereby an EC undergoes a series of molecular events that lead to a change in phenotype toward a mesenchymal cell. In a hypothesis and theory article, Fang et al. propose that an EndoMT program is partially and reversibly activated event in angiogenic ECs to support acquisition of the subset of mesenchymal characteristics, which is necessary to develop sprouting angiogenesis. They also discuss the potential signaling and regulatory mechanisms that may control the EndoMT program as well as potential therapeutic approaches in cancers. EndoMT is known to be regulated by transforming growth factor-β (TGF-β) family. Ma et al. report that TGF-β2 may be essential for this process by regulation of the balance between transcription factor SNAIL and ID factors (Ma et al.) and this may also regulate angiogenesis (Akil et al.; Fang et al.). Additionally, a study by Hunyenyiwa et al. shows that obesity inhibits angiogenesis via TWIST1-SLIT2 signaling, similar to an angiogenic phenotype occurred in tumor angiogenesis under diet-induced obesity conditions (Dong et al., 2017). It should be noted that Twist 1 is known as a master transcription factor of EndoMT. The study thus suggests that obesity may determine the angiogenic fate via regulation of the EndoMT. Interestingly, Sun et al. reveal that ROS and RBC phosphatidylserine exposure can mediate brain endothelial erythrophagocytosis, implicating in cerebral microhemorrhage-like lesions independent of disruption of the microvasculature.

In addition to new sprouting from blood vessels, the sprouting of lymphatic vessels, lymphangiogenesis, is actively involved in many pathological processes including tissue inflammation and tumor dissemination but is insufficient in patients suffering from lymphedema. Cilia, a microtubule-based organelle expressed on the apical surface of blood ECs is thought to function as a blood flow sensor. Paulson et al. show that primary cilia exists in lymphatic vasculature, and surprisingly is expressed on the abluminal surface of the lymphatic vessel. They also present evidence that lymphatic vessel patterning is regulated by a primary cilium protein IFT20 in lymphatic ECs during development and inflammation, which could lead to a new paradigm in the field of lymphangiogenesis. Last but not the least, Norden and Kume provide an elegant review about current status and the mechanisms by which lymphatic permeability and function are regulated in a tissue- and organ-specific manner, including lacteals of the small intestine. Disrupted lymphatic EC junctions are associated with various diseases, including lymphatic leakage present in chylothorax and lymphedema, metabolic syndrome, and impaired immune surveillance. They also describe key signaling pathways and factors that control lymphatic EC junctional integrity including VEGF, S1P, LPA signaling molecules and FOXC1 and FOXC2 transcription factors as well as RhoA/ROCK and Ephrinb2-Ephb4 pathways.

## Summary and Prospective

Heterogeneous endothelium and vasculature play a pivotal role in the regulation of tissue and organ homeostasis in human health and disease progression. This topic only covers the tip of iceberg in the field of EC and vascular biology, and significant studies are urgently needed to move the field forward. One emerging area is the immunological properties of ECs and their role in angiogenesis and lymphangiogenesis. ECs show distinguished immunological functions and have innate and trained immunity (Shao et al., 2020; Drummer et al., 2021; McCoy et al., 2021). McCoy et al. recently demonstrate that ECs facilitate proangiogenic immune cell recruitment and contribute to tumor angiogenesis *via* toll-like receptor 2 signaling (McCoy et al., 2021). Secondly, angiogenesis is regulated by paracrine and autocrine mechanism, including regulation by VEGF and hypoxia-inducible factor 1α (HIF1-α) whereas HIF-α directly regulates VEGF under ischemic conditions, thereby leading to angiogenesis and arteriogenesis in ischemic diseases and cancers (Semenza, 2007; Fong, 2009; Ren et al., 2010; Ho et al., 2012; Ren, 2016; Simons et al., 2016). The resulted neovascularization may improve ischemic conditions or promote tumor progression via provision of CSCs with a favorable vascular microenvironment (Jiang et al., 2021). Thirdly, the studies on EC differentiation and transdifferentiation are emerging as an important area, and they contribute significantly to capillary arterialization or *de novo* arteriogenesis, particularly under pathological conditions (Mac Gabhann and Peirce, 2010; Ren et al., 2010; Moraes et al., 2013; Ren et al., 2016a; Ren et al., 2016b; Ren et al.; Jiang et al., 2021). Furthermore, venous ECs may contribute to the generation of arterial ECs and expansion of arteriolar networks (Lee et al., 2021). Key pathways and gene signature may be conserved in certain types of ECs (Ren et al., 2011; Coppiello et al., 2015; Ren et al., 2016a; Jiang et al., 2021) but it would be of significance to study EC biology and angiogenic and lymphangiogenic processes using heterogeneous types of ECs, animal models and human patient samples before novel findings and concepts can be effectively translated into clinic settings.
